# Metagenomic mining and structure-function studies of a hyper-thermostable cellobiohydrolase from hot spring sediment

**DOI:** 10.1038/s42003-022-03195-1

**Published:** 2022-03-22

**Authors:** Migiwa Takeda, Seiki Baba, Jiro Okuma, Yoshitsugu Hirose, Asuka Nishimura, Masaki Takata, Kohei Oda, Daisuke Shibata, Takashi Kumasaka, Yasuhiro Kondo

**Affiliations:** 1 Honda Research Institute Japan Co. Ltd., Kazusa Incubation Center, 2-1-5 Kazusa Kamatari, Kisarazu, Chiba 292-0818 Japan; 2 Japan Synchrotron Radiation Research Institute (JASRI), SPring-8, 1-1-1 Kouto, Sayo, Hyogo 679-5148 Japan; 3 RIKEN SPring-8 Center, 1-1-1 Kouto, Sayo, Hyogo 679-4198 Japan; 4 Kazusa DNA Research Institute, 2-6-7 Kazusa-kamatari, Kisarazu, Chiba 292-0812 Japan; 5 Present Address: Innovative Research Excellence, Power Unit & Energy, Honda R&D Co., Ltd., 1-4-1 Chuo, Wako, Saitama 351-0193 Japan; 6 Present Address: Graduate School of Agricultural and Life Sciences, The University of Tokyo, 1-1-1 Yayoi, Bunkyo-ku, Tokyo 113-8657 Japan; 7 Present Address: International Center for Synchrotron Radiation Innovation Smart, Tohoku University, 2-1-1 Katahira, Aoba-ku, Sendai 980-8577 Japan

**Keywords:** Environmental biotechnology, X-ray crystallography

## Abstract

Enzymatic breakdown is an attractive cellulose utilisation method with a low environmental load. Its high temperature operation could promote saccharification and lower contamination risk. Here we report a hyper-thermostable cellobiohydrolase (CBH), named HmCel6A and its variant HmCel6A-3SNP that were isolated metagenomically from hot spring sediments and expressed in *Escherichia coli*. They are classified into glycoside hydrolases family 6 (GH6). HmCel6A-3SNP had three amino acid replacements to HmCel6A (P88S/L230F/F414S) and the optimum temperature at 95 °C, while HmCel6A did it at 75 °C. Crystal structure showed conserved features among GH6, a (β/α)_8_-barrel core and catalytic residues, and resembles TfCel6B, a bacterial CBH II of *Thermobifida fusca*, that had optimum temperature at 60 °C. From structure-function studies, we discuss unique structural features that allow the enzyme to reach its high thermostability level, such as abundance of hydrophobic and charge-charge interactions, characteristic metal bindings and disulphide bonds. Moreover, structure and surface plasmon resonance analysis with oligosaccharides suggested that the contribution of an additional tryptophan located at the tunnel entrance could aid in substrate recognition and thermostability. These results may help to design efficient enzymes and saccharification methods for cellulose working at high temperatures.

## Abstract (web summary)

Bacteria from hot springs are known for highly thermostable enzymes, which may have industrial potential. Here, a unique thermostable cellobiohydrolase is reported that can breakdown cellulose at temperature up to 95 degrees Celsius.

## Introduction

Cellulose is a major component of plant cell walls, and the most abundant, renewable carbon material on Earth. As a basic ingredient of second-generation biofuel, its conversion to sugar has received extensive attention in the biorefinery industry^1–4^. In comparison with its chemical hydrolysis, biological enzymatic processing is preferable due to small environmental load. In nature, one of the well-studied wood-decay fungi, *Hypocrea jecorina*, aka *Trichoderma reesei* can perform to degrade plant cell walls via synergistic reactions of secreted glycoside hydrolases (GHs), which at least include endoglucanases (EGs), cellobiohydrolases (CBHs) and β-glucosidases (BGs)^5,6^. To mimic the processing in vitro, enzyme cocktails mainly composed of these enzymes have been extensively studied and some of them are successfully applied in practical use^7^. These studies revealed that it is favourable for the enzymes to have three key features: high catalytic efficiency, high thermal stability, and low end-product inhibition. Indeed, high enzyme load is sometimes required to increase saccharide yield but also enzyme cost due to the limited lifespan of the enzymes originating from mesophilic organisms^4^. As a strategy to resolve the issue, the enzymatic cocktails for higher temperature have been proposed^8,9^. Thermophilic enzymes exhibit many useful features for this purpose, such as a longer biocatalyst lifespan, a faster catalytic rate^10,11^ and a lower product inhibition at higher temperatures^12^. Moreover, the high-temperature processing has advantages, such as preventing the growth of contaminated harmful bacteria or lowering the viscosity in the reaction mixture to save operation energy^13,14^. For these reasons, one of the challenges is to achieve thermophilic cocktails working at the highest possible temperature, such as above 90 °C, where hyperthermophiles live^8,15–17^.

Thermostable proteins have long been investigated but are continuously advancing by incorporating various technologies such as protein engineering and enzyme discovery from metagenomes^18^. The rational engineering approach expects to incorporate structural features that contribute to thermostability, such as surface charge distributions^19^ and structural elements (hydrogen bonds, disulphide bridges, metal bindings, and loop stability). Directed evolution techniques can also be applied to select characteristic enzymes from gene libraries. However, both methods generally limit the seeking range of chemical space in polypeptides depending on the variation of artificial designs or the screening capability^7,18^. Therefore, the discovery of extraordinary enzymes from natural environments remains important. In addition to traditional isolation and cultivation methods for extremophiles, the metagenomic approach is currently used to survey genes that have evolved to survive under extreme environmental conditions, such as hot springs and deep-sea hydrothermal vents^20^. Recent advances in sequencing technology have made it possible to obtain large amounts of data directly from environmental DNA samples, even from viable but non-culturable microorganisms. Therefore, sequencing of DNA samples from high-temperature environments may help find unknown thermostable enzymes, as shown in early studies on the termite hindgut^21^ and cow rumen^22^.

Currently characterised CBHs are still inadequate for achieving the hyper-thermostable cocktails, even in the many efforts for enzyme isolation from thermophilic organisms^23,24^ and engineering of mesophilic enzymes raising the optimum operating temperature^25–29^. In other words, the natural hyperthermophilic CBHs have not yet been isolated, while several EGs and BGs have been isolated from hyperthermophilic archaea and bacteria^9,15,17^. CBH is thought to be evolved from EG by an addition of accessory structural loops forming a substrate-binding tunnel for achieving exo-acting hydrolysis^30^. Therefore, CBHs are mostly categorised into GH families 5, 6, 7, 9 and 48 together with EGs, and further classified into two types by acting ends of cellulose: CBH I acts at the reducing end of cellulose and belongs primarily to GH 7 and 48, while CBH II acts at the non-reducing end and belongs to GH 6. This exo-acting reaction releases cellobiose units from the terminal end of a cellulose chain, through a process that involves sequential catalytic steps without dissociating from the polymer^31,32^. Since CBH is easily captured to insoluble cellulose, this reaction sets the rate limit during the synergistic hydrolysis and then higher enzyme amounts are required for efficiency^31^. Moreover, the stability of the tunnel-forming loops and the affinity of subsites at the exit site of the reaction product tend to cause thermostable difficulty in stability-function tradeoffs and product inhibition of the enzymes, respectively^33–35^. Due to these discriminative features, CBH is the key enzyme in achieving the hyper-thermostable cocktails.

Here we report a highly-thermostable GH 6 CBH II and its variants obtained by a metagenomic approach from a hot spring sediment. The enzymes were from a bacterial origin and well expressed with *E. coli*, as well as characterised through crystallographic and enzymatic analysis. The enzyme displayed the highest thermostability over 95 °C among the currently known CBHs.

## Results and discussion

### Metagenomic analysis of hot spring sediment

We employed a sequencing-based metagenomics approach to mine CBH genes from environmental DNA that was isolated from hot spring sediments in Miyagi prefecture, Japan. The first DNA sample, named AR19, was sequenced in triplicate using 454 pyrosequencing, which included a total of 2,766,332 reads, with an average sequence length of 400 ± 55 bp, totalling 1.1 Gbp of sequencing data (Table S1). Of these, 17,991,567 reads (68.4%) were assembled into contigs ≥1 kb (595,602 contigs). The largest contig was 278,185 bp. Phylogenetic binning of all contigs and singletons in AR19 was performed using BLAST, then compared to the KEGG database^36^ to classify the data into bacterial, archaeal, eukaryotic, viral or unclassified sets. Contigs and singletons classified as bacteria and archaea accounted for 59.9 and 3.0 Mbp, respectively, whereas the unclassified set was 266.7 Mbp, which suggests that most (80.8%) of the obtained sequences from AR19 were unknown. Eukaryotic and viral sequences made only minor contributions.

Table S2 shows the carbohydrate-active enzyme (CAZy) annotation of cellulases predicted to have a high significance (E-value < 1 × 10^−5^) to correspond to an enzyme in the CAZy database^5^. The table also shows the CAZy family-associated protein domain (Pfam) annotation. Overall, we predicted the presence of 3378 GHs (1.96% of all open reading frames (ORFs)). A total of 75 ORFs were identified as putative cellulases (endoglucanases and cellobiohydrolases) belonging to the families GH5, GH6, GH9, GH44 and GH48. This corresponds to 6.1% of all GH enzymes. Among them, 70 ORFs, except for GH44, corresponded to 5.7% of all GHs and are considered CBH candidates.

DNA sequences of the 70 ORFs were amplified from the environmental DNA using PCR with specific primers designed for each ORF and cloned into the vectors of *E. coli*. Since the contig sequences were a mixture of closely resembled sequences that existed in the environmental DNA, a cloned sequence of a particular ORF may not be identical to the sequence predicted from the assembled contigs. Thus, we sequenced at least 10 clones for each ORF to confirm the sequences. In most cases, multiple genetic variants were identified for each ORF.

### Enzyme characterisation

The DNA sequences that were predicted as CBHs and their genetic variants were expressed in *E. coli*. Enzymatic assays of hydrolase activity with phosphoric acid-swollen Avicel (PSA) were then performed. Most showed no or very weak activity toward the substrate, which coincides with reports that the expression of active CBHs is difficult in *E. coli*^37^, likely due to there being no proper assembly of the proteins in the host. As a result, none of the CBH candidates belonging to GH families 5, 9 or 48 were PCR-cloned or expressed in *E. coli*. Nevertheless, two GH6 CBH genes showed significant activities, and were eventually identified and cloned for heterologous expression in *E. coli*. The observed activity was also confirmed using crystalline cellulose Avicel. The catalytic domains of the two genes shared a high degree of amino acid sequence identities (80% identity over 344 equivalent residues).

**Fig. 1:**
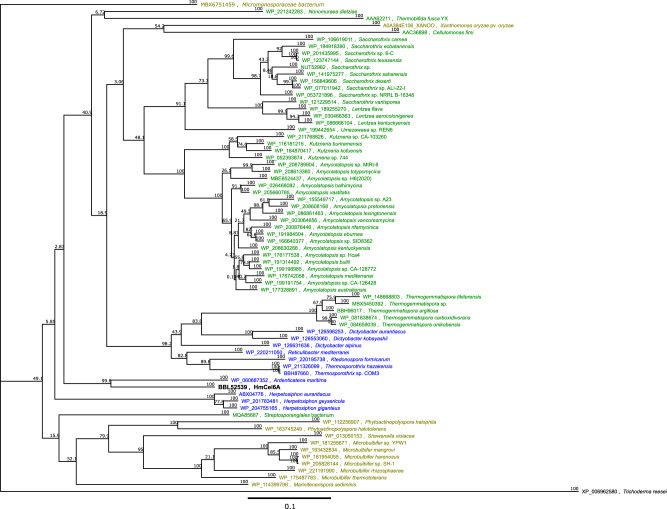
Non-rooted phylogenetic tree of bacterial GH family 6. Bootstrap values at branch points are indicated for 10,000 replicates and shown as percentages. Scale bar = 0.1 amino acid substitutions per site. Branches corresponding to partitions that were reproduced in <50% of bootstrap replicates are collapsed. The tree is drawn to scale, with branch lengths in the same units as those of the evolutionary distances that were used for phylogenetic tree construction. The sequences of three bacterial structure-determined CBHs and CBH II from *Trichoderma reesei* (*Hypocrea jacorina*) were also included. Species belonging to high G + C Gram-positive bacteria, green non-sulfur bacteria and γ-proteobacteria are colorised with green, blue and olive, respectively.

Among these, a GH6-family hydrolase, named HmCel6A (hot spring metagenome-derived cellulase family 6A), showed the highest activity. Thus, we focused thereafter on HmCel6A and its genetic variants. The genes seemed to encode a full-length catalytic domain as a CBH. Homology searches against multiple databases showed that HmCel6A shared amino acid sequences that were 76% similar to the GH6 catalytic domain sequence from *Ardenticatena maritima*, a ferric iron- and nitrate-reducing bacterium belonging to the phylum Chloroflexi^38^. The phylogenetic tree suggests that this CBH is of bacterial origin (Fig. 1).

**Fig. 2:**
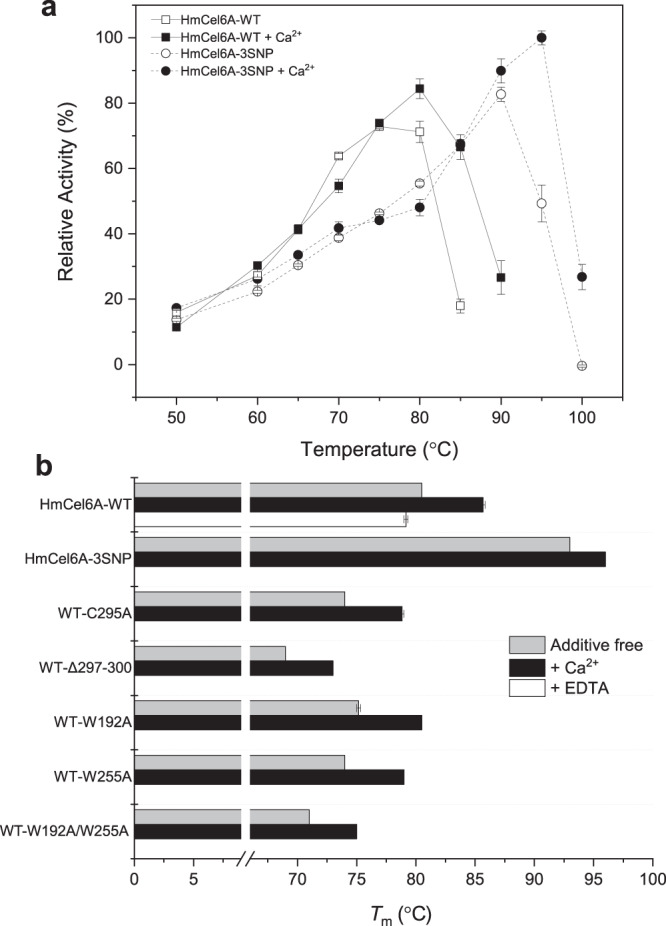
Enzymatic properties of HmCel6A. **a** Optimum temperature (*T*_opt_) of HmCel6A and its 3SNP variant with the PSA substrate in the presence (solid squares and circles) and absence (white squares and circles) of 3 mM CaCl_2_. **b** Melting temperature (*T*_m_) of wild-type and mutant enzymes in the presence (black bars) and absence (grey bars) of 3 mM Ca^2+^/40 mM EDTA, as determined in a thermal shift assay. Each error bar represents the standard error.

This recombinant HmCel6A showed hydrolytic activity against the crystalline cellulose Avicel and PSA (Table S3). The optimum temperature (*T*_opt_) and the melting temperature (*T*_m_) for PSA were 75 °C and 80 °C, respectively, at the optimum pH of 5.5 (Figs. 2a, b and S1). The addition of calcium ion to the reaction mixture improved the thermostability, as seen in a thermal shift assay^39^ (Fig. 2b). Further metagenomic analysis included the identification and activity characterisation of 12 genetic variants of HmCel6A (Table S4). HmCel6A-3SNP, isolated from the metagenomic sample OSJ2, had three amino acid replacements to HmCel6A (P88S/L230F/F414S), exhibited the highest *T*_opt_ of 95 °C with PSA as a substrate (Fig. 2a) and had a *T*_m_ of 96.0 °C in the presence of calcium (Fig. 2b). This provided us the unique opportunity to investigate the effect of amino acid residues on thermostability.

### Overall structure of HmCel6A

**Fig. 3:**
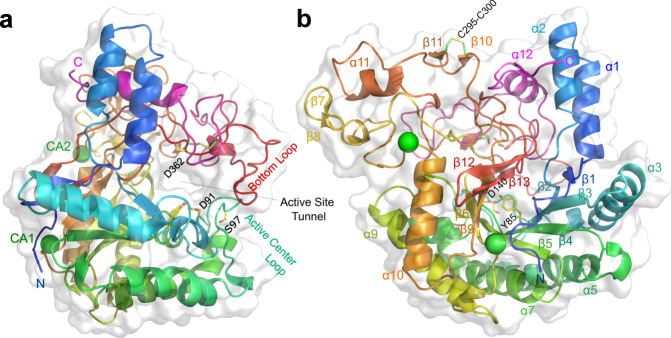
Crystal structure of HmCel6A. **a**, **b** Two views (in ribbon representation) of the Ca^2+^-bound structure. The protein chain is blue to red from the N- to C-terminus. Calcium ions, CA1 and CA2, are displayed as green spheres. The active site is enclosed in a tunnel formed by interactions between the extended bottom loop and the active site loop. Catalytically important residues and disulphide bonds are shown in stick representation. **a** Ribbon representation of HmCel6A overlaid on the Connolly surface representation. **b** View showing the β/α barrel structure with a central β-barrel comprising nine numbered strands.

The crystal structure showed a (β/α)_8_-barrel core (Figs. 3, S2a), and putative catalytic residues, such as Asp140 of the catalytic acid, that are generally conserved among the GH6 enzymes^23,40,41^. Although GH6 includes EG and CBH II, all the GH6 CBHs shared the active-site loop and the extended bottom loop, which formed the active-site tunnel^23^ (Fig. S2b). These are also known as the N-terminal and C-terminal loops in fungal enzymes. The structure of HmCel6A is more similar to three bacterial CBH II enzymes, TfCel6B from a soil cellulolytic actinomycete *Thermobifida fusca*^23^, CfCel6B from a cellulolytic facultative anaerobes *Cellulomonas fimi*^42^ and XooCbsA from a phytopathogenic bacterium *Xanthomonas oryzae* pv. *oryzae*^43^. In particular, the three loops located around the substrate entry and exit sites were common and characteristic among the bacterial enzymes.

On the other hand, we identified several unique characteristics of this enzyme that presumably contribute to thermostability. As global properties, HmCel6A is rich in hydrophobic clusters and charge–charge interactions (Table S5). Hydrophobic clusters are mostly observed in the major lobe of the GH6 enzymes. HmCel6A has the largest dimensions of overlapping area, and HmCel6A-3SNP has the largest cluster consisting of 145 contacts among all the known GH6 CBH structures. The charge interactions were shown in an increased number of salt bridges and as the lowest free energy in protein charge–charge interactions formulated with the Tanford–Kirkwood Surface Accessibility (TKSA) model^19^, which accounts for the effects of solvent polarization on charged atoms in proteins. The numbers of hydrogen-bonds are not significant but specific hydrogen bond networks are observed. Further, we found several structural elements for thermostability: an additional calcium ion, a disulphide bond located on the protein surface, interactions between the active-site loop and the bottom loop, and two shortened loops located at the substrate entry and exit sites (Fig. 3). Some details are described in the next section.

### Key structural elements for thermostability

One unique structural feature is calcium binding. Unlike the fungal GH6 CBHs, HmCel6A as a bacterial enzyme has metal-binding sites, whose elements were identified as calcium ions contained in the crystallization condition. In addition to the CA1 site shared with TfCel6B (Fig. S2d), a unique metal-binding site (CA2) is located on the loop between β6 and α1 (Fig. S2c). The effect of calcium was experimentally verified by adding calcium salt to the enzyme solution, which enhanced its thermostability (Fig. 2); *T*_opt_ with PSA was 75 °C and 80 °C in the absence and presence of 3 mM calcium, respectively, and *T*_m_ was 80.5 °C and 85.5 °C, respectively. In the crystal structure of the 3SNP variant, neither metal ion was observed, as the crystals were grown in metal-free solution, but the effect of calcium on the enzyme activity was retained. The effect of other metal ions was also examined as shown in Fig. S3. We could not observe improvement of *T*_opt_, but manganese-enhanced enzyme activity rather than calcium, and ferric and zinc ion reduced the activity in this condition. This result is almost same with previous reports for other CBHs^44,45^.

Another distinctive feature of HmCel6A is its disulphide bonds. The two disulphide bonds observed in the crystal structure of HmCel6A (Cys92-Cys154 and Cys331-Cys383) are typically found in GH6 CBHs^23,40,41^, and presumably stabilise the tunnel-forming loops. A third additional bond (Cys295-Cys300) in HmCel6A forms a short ring structure consisting of six residues. This additional bond is not present in other GH6 CBHs (Fig. S4). The ring fills a cavity in the molecular surface, and engages in interactions with other structural elements; thereby, possibly contributing to the enzyme’s structural stabilisation (Fig. S2e). Indeed, when the ring was opened by the C295A mutation, *T*_m_ was decreased by 6.5 °C, and deletion of the ring itself further decreased *T*_m_ by 11.5 °C (Fig. 2b).

Three mutations in the highest thermostable 3SNP variant only affected the local structure, although some hydrophobic interactions were replaced by charged interactions relative to the wild-type enzyme. Phe414 located in a hydrophobic core was replaced with Ser to introduce the Trp409-Gln23-Glu415-Thr27 hydrogen bond network at the molecular surface. This replacement was the most effective from the three mutations, since it led to an increase in *T*_m_ via a single mutation. Ser88 introduced an intramolecular water molecule and might compensate cavity around the residue. Phe230 might incorporate π-π and/or anion-π interactions with the neighbouring residues, Tyr352 and Glu231. Together, these structural features appear to improve the thermostability of HmCel6A, and could be engineered into other GH6 enzymes; however, this replacement reduced the relative activity to 20%–30% at *T*_opt_ of wild-type (WT) (Fig. 2a).

### Structural basis of catalytic cycle

The catalytic cycle in GH6 CBHs consists of four modes: pre-slide mode, slide mode, Michaelis complex and substrate-product complex^35^. We identified three modes in HmCel6A, but were unable to identify the slide mode using the crystal structures. The mobility of the well-conserved active-site loop and its open/close flexibility is thought to contribute to its processive hydrolysis, in order to rotate the catalytic cycle. Ser97, the key residue for the motion, forms a hydrogen bond with the main chain atoms of Gly99, and the proton-acceptor Asp222, in open conformation and is moved toward the subsite −1 in close conformation after the substrate slides to subsite −1 and −2^35^.

The pre-slide mode was identified by the complexing of the crystal structure’s chains B and C with cellotriose (Glc3). In this complex, the substrate only occupied the +1 to +3 subsites and each glucose moiety was similar to those observed in other GH6 enzymes, with their ^4^C_1_ conformation. The active-site loop took on the open conformation. In HmCel6A, Ser97 uniquely formed a hydrogen bond at its main chain carbonyl with Lys378 Nε located in the bottom loop. Thus, the active-site loop slightly opened to the solvent region, in the so-called ‘even more open’ conformation.

The Michaelis complex mode was observed when the crystal complexed between cellohexaose (Glc6) and the inactivated enzyme, which mutated at the catalytic acid residue Asp140Ala. The substrate occupied subsites −3 to +4, with the partial occupation of both its ends. The ligand binding affected the active-site loop, in which Asn98 side chain was in close form. The puckering conformation of the glucose moiety was ^2^S_O_ at the −1 subsite. This was well observed, since it played a central role in the activation of the substrate and product expulsion.

**Fig. 4:**
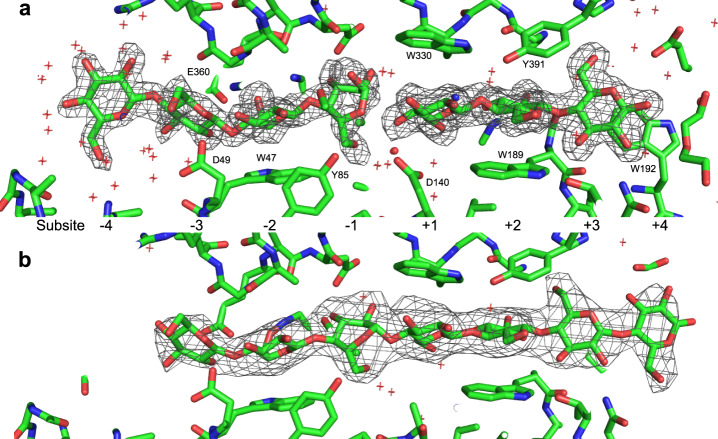
Structures of oligosaccharide binding to HmCel6A. **a** Glc3 oligomers bind to wild-type enzyme. **b** Glc6 oligomer binds to D140A mutant of HmCel6A. Electron density was calculated as omit Fo-Fc map and contoured with 3σ.

The substrate-product complex was obtained as the structure of chain A in the Glc3 complex, in which one cellotriose bound to subsites +1 to +3, and another cellotriose bound to two binding modes at either subsites −4 to −2, or subsites −3 to −1 (Fig. 4). Even though the electron density at the −1 subsite fluctuated by partial occupancy, it seemed to digest the covalent bond between +1 and −1, and to take a skew-boat conformation (^2^H_1_ or ^2^E: *φ* = 105.421°, *θ* = 50.914°, *Q* = 0.682). This conformation was unlike the chair conformation (^2^S_O_), but similar to ^2,5^B, as observed in HiCel6A when complexed with a cellobiose derivative^46^. The broad electron density around the O1 atom, and the residual electron density around the C1 atoms were considered to partly include the Michaelis complex (Fig. S5a).

While the active-site loop is generally indispensable to catalysis in the GH6 CBH II enzymes, the bottom loop might only contribute the tunnel formation. Its sequences are diverse and provisionally categorised into seven groups^47^. The bottom loop of HmCel6A could not be assigned into any of these groups, and we found some unique structural features regarding its rigidity: (i) it uniquely contained three prolines (QPGIVDPDDPNKK), (ii) it had Lys378, which maintained the open conformation of the active-site loop and (iii) it had three ionic interactions (Asp371 formed a salt-bridge with Arg408, the main chain carbonyl of Asp373 joined with Arg50, and Asp374 joined with Lys377). Nonetheless, the bottom loop moved cooperatively to close the active-site loop, and introduced some hydrogen bonds to fixate the reaction intermediates such as Arg50-Asp374, Asn198-Lys378, Asp380-Ala95 NH2, Asn376-Gly99 CO and Arg90-Ser97 CO.

The smooth expulsion of the reaction product from each subsite is essential to avoid product inhibition and to obtain the highest enzyme efficiency. GH6 CBH enzymes might have originally evolved from EG, which has created high binding affinity at subsites −3 and −4 that enhances to stay the end product there. TfCel6A introduces the extended exit loop, as a “gatekeeper,” which largely moves to the region via substrate binding but without any direct interaction with the saccharide at subsite −2^47^. Most fungal enzymes have no exit loop, so the product binding cannot be ignored as observed in a crystal structure of HiCel6A, PDB-ID 1OCB (24). HmCel6A has a short exit loop fixated with a salt-bridge between Arg58 in the loop and Asp15 in the α1 helix of the β/α barrel core, thus its mobility and gatekeeper role might be lost. Asp49, Arg50 and Glu360 possibly contribute saccharide binding at the subsite −3. All these residues are found in TfCel6A, and the aspartate and the glutamate are also observed in a bacterial EG, TfCel6B. To observe the role of the extended exit loop in TfCel6A, its insertion in HmCel6A was examined. The extension tended to cause activity reduction (Fig. S6), which might relate to product inhibition at higher temperatures, but further investigation, is required.

### Characteristics of substrate recognition

**Fig. 5:**
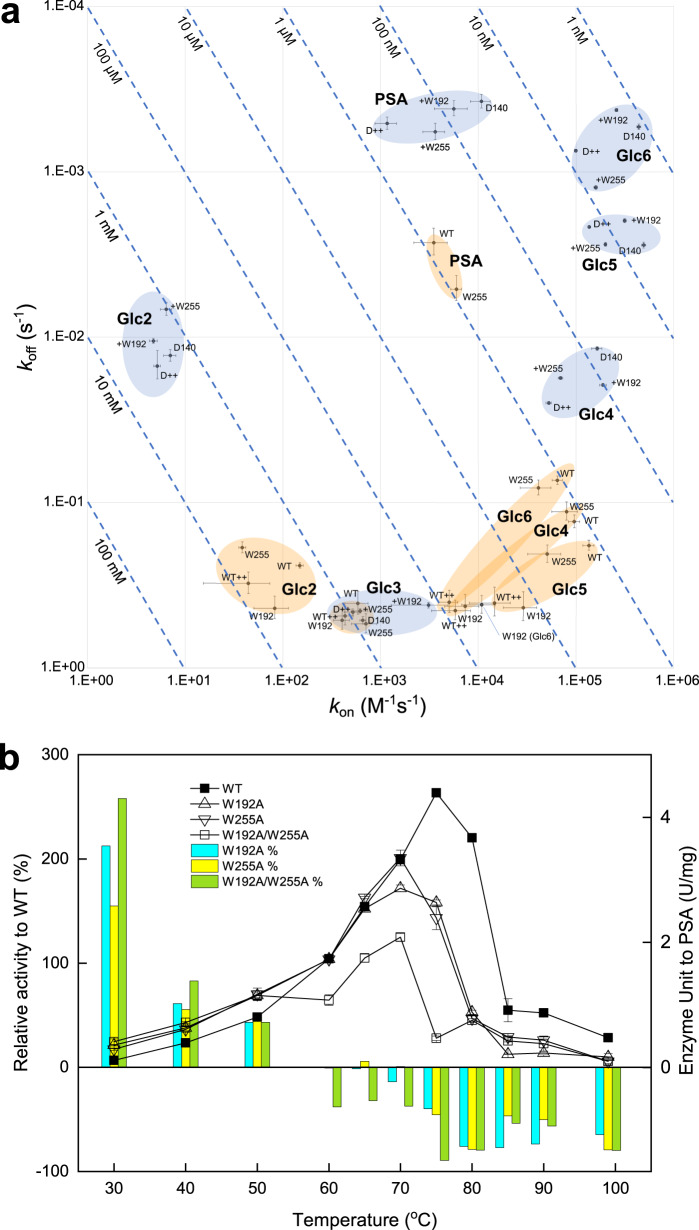
Effects of W192 and W255 mutation in HmCel6A. **a** SPR *k*_on_-*k*_off_ plot for various degrees of polymerisation in substrates. Cyan and orange ellipsoids correspond to being with and without catalytically deficient mutations (D140A), respectively. Each error bar represents the standard error. **b** Enzyme activity against temperature. Relative activity against the wild-type enzyme and enzyme unit are drawn in bar and line, respectively. Each of the two tryptophans affect turnover at lower temperatures and enzyme stability at higher temperatures.

As described above, the substrate recognition scheme of HmCel6A is almost the same as other GH6 CBHs. We analysed the degree of polymerisation for substrate against the WT and catalytically deficient D140A mutants using the surface plasmon response (Fig. 5a), in order to reveal more detail of the HmCel6A substrate recognition scheme. The results of the WT enzyme might underestimate the dissociation constant rate (*k*_off_) given the existence of the product binding. Cellobiose (Glc2) and Glc3 were not hydrolysed or were hydrolysed quite slowly by the enzyme. PSA and cellotetraose (Glc4) or longer substrates can thus be hydrolysed, and product binding can occur similar to Glc2 and Glc3, which may act as the product.

Glc2 is not hydrolysed even in the catalytic enzymes, therefore its affinities directly relate to product inhibition. Its affinities for WT and D140 were almost identical. This is reasonable, since almost all the substrates stayed off of the subsite −1 in the reported crystal structures of catalytic GH6 CBH, where Glc2 was mostly between the +1 and +2 subsites^41^. Of course, Asp140 contributes to substrate recognition at the +1 binding subsite, therefore the affinity at subsite +1 might be lowered in the mutant. On the other hand, subsites −1 or +1 generally have a lower affinity, because the saccharide ring distorts to a conformation that is energetically unfavourable for the activation of the scissile bond. The other sites, especially the −2 or +2 subsites, adapt short chains and harness the sugar chain to be fixated for activity^35^. Glc3, to which HmCel6A exhibits almost ignorable catalytic activity, showed a slightly higher affinity due to the increased interactions of one added sugar with the enzyme. In fact, the structure of the Glc3-complex in the WT showed that Glc3 was found at subsites +1 to +3, as described above. This binding mechanism at the exit site might not be directly related to the product inhibition by Glc2. If this ligand came from not the entry side of the substrate tunnel but its exit side, the binding of Glc2 at subsites +2 and +1 requires that Glc2 occupy subsites +3 and +2 first and then not only slide but also rotate.

Total affinity increased with length in the longer and hydrolysable oligomers from Glc4 to Glc6, as clearly observed in the results from the inactivated D140A mutant. As described above, the −2 and +2 subsites may predominantly play a role in the affinity of the enzyme, since tetra-saccharide has a stronger affinity than the shorter oligomers. Of course, even if the D140A mutation inactivates an enzyme, it might affect to binding affinity at the −1 or +1 subsites. In fact, the sugar was skewed at the −1 subsite of our Glc6 complex crystal structure. An important residue to maintain this skewing is Tyr169 in TrCel6A and Tyr85 in HmCel6A, because its phenylalanine replacement introduces space around the catalytic site and reduces the constriction of the sugar^40^. Similarly, the Asp140 short side chain that flanks the tyrosine might somewhat increase the affinity of the −1 and +1 subsites using the same mechanism. The WT enzyme showed mostly similar binding affinities for all these oligomers, which gradually degrade during measurement. The *k*_off_ values produced shorter chains, while the *k*_on_ values were maintained, despite being impacted by the shorter chains. The PSA *k*_off_ value was slightly higher than that of Glc6 in the D140A mutant. It is unclear if the difference was caused by the presence of additional subsites. We concluded, that the differences in the *k*_on_ values depend on the crystallinity of these substrates.

### Effects of tryptophan on catalysis

Tryptophan is a well-observed residue that supports saccharide binding in GHs. Its effect has been confirmed in both CBH I and II^48^. In HmCel6A, there are five tryptophans around the active-site tunnel (Trp47, Trp189, Trp192, Trp255 and Trp330). Among them, we focused Trp192 and Trp255 located on the entry side of the tunnel to confirm the further catalysis details (Fig. S5b). Its reason is that the cellulose chain binding at entry side initiates enzymatic processing and then both residues might determine the ability to capture the substrate. Trp192 is conserved well in CBH II and forms the subsite +4^49,50^, while Trp255 is observed in bacterial CBHs and seems to bait substrates, such as an additional subsite +6^23^. We constructed catalytic and catalytically deficient D140A mutants for both residues, then further analysed them using an enzyme assay and the surface plasmon response (Fig. 5).

Both mutations totally reduce affinity, which indicates that the tryptophans contributed to substrate recognition. In fact, the affinity for non-hydrolysable Glc2 was reduced in both mutants of the catalytic enzyme (Fig. 5a), which might simply describe its contribution to the affinity. A similar SPR experiment for a GH18 chitinase showed that tryptophan mutations consisting of +2 or −3 subsites lost 8.2 or 5.7 kJ/mol, as calculated from *K*_d_ for (GlcNAc)_4_^51^. Our inactivated mutants did not show any significant differences. The reason for this is not clear, but may be due to compensation for the affinity gained by the D140A mutation at the +1 and −1 subsites, as described above.

The *k*_on_ values decreased for Glc4 to 6 in the Trp192 mutations of the catalytic enzyme (Fig. 5a). In fact, both of the crystal structures complexed with cello-oligomers and showed highly-occupied +2 and +3 subsites, thus, Trp192 might contribute to the binding of Glc4 to 6. This is quite similar to how the relevant mutation in Trp332 of TfCel6B decreased *K*_d_ in 20–100-folds that were analysed by fluorescence titration^50^. For polymeric substrates, the mutation increased activity in PSA at 30–50 °C, as shown in Fig. 5b, since PSA is a soluble polymer with rather similar characteristics to soluble cello-oligomers. A similar phenomenon was reported in the relevant Trp272 mutation of TrCel6A, which caused an increase turnover (*k*_cat_) in cello-oligomers. This is explained by the removal of some non-productive binding mechanisms, which prolonged the retaining period of the substrate^49^. In Avicel, the HmCel6A mutant decreased in activity, which could be explained by the reduced absorption of the substrate at higher temperatures (Fig. S7). Similarly, in TfCel6A, the relevant mutation impairs the enzyme’s function against bacterial microcrystalline cellulose (BMCC)^50^. These results suggest that Trp192, which is located near the entrance of the active-site tunnel, may assist in the hydrolysis of crystalline cellulose by helping a substrate chain enter the active site.

Trp255 is located farther from the active centre than Trp192 with a limited energy gain for soluble oligosaccharides. In fact, a low but significant effect on affinity was observed (Fig. 5a). Nevertheless, the affinity and activity for the polymeric substrate reduced by the same amount as the Trp192 mutation. Furthermore, the double mutation of Trp192 and Trp255 caused an additional decrease. It has been inferred that the relevant residue, Trp394, could form a +6 subsite in TfCel6A^23^. In addition, the Trp394 residue has a stronger affinity for longer substrates^23^. It has also been argued that its previous residue, Asp393, corresponded to Glu254 in our structure. The relevant residues to the pair of acidic amino acid and tryptophan are also observed in Cel6C, which is from the basidiomycete, *Coprinopsis cinereal*^52^. In the binding model of TfCel6B to crystalline cellulose, Trp332/Trp192 supports a cellulose fibre pulled from the crystalline structure in order to introduce it into the active-site tunnel^47^, while Trp394/Trp255 may maintain its interaction with the surface of the crystalline. These residues may have similar roles in CBH I. Trp40 in TrCel7A, a CBH I in GH7, forms the subsite −7 at the entrance edge of the active-site tunnel, and is thought to initiate the degradation of crystalline cellulose^48^, even though +6 subsite of GH6 CBH II is exposed to the solvent differently.

In addition, we observed that both tryptophans contributed to the thermostability of the enzyme. The alanine mutants for W192, W255 and both showed a *T*_m_ of 75.2, 74.0 and 71.0 °C, respectively, which are 9.5 °C lower than the WT. Further investigation of these residues in the high-temperature enzymatic saccharification process is warranted, since high temperatures cause changes in the protein energy landscape.

### Conclusions

The newly identified cellobiohydrolase, HmCel6A, can be expressed in the heterologous host *E. coli*. A variant of HmCel6A displayed its highest optimum temperature at 95 °C. This enzyme has unique structural features, such as metal binding, disulphide bonds and shortened loops around the substrate tunnel, in which the bottom loop has a novel sequence. An additional tryptophan, Trp255, is located at the enzyme’s tunnel entrance, and might contribute to catalysis and thermostability. With these features, this enzyme may contribute to the establishment of an efficient, high-temperature saccharification process for cellulose, which may allow for large-scale, industrial use. Indirectly, these features can help to improve CBH II via protein engineering techniques.

## Methods

### Nucleic acid extraction

The sediment samples were collected from Onikobe-Jigokudani geothermal area in Miyagi, Japan in 2009 and 2012 (Table S4). The samples were immediately placed on ice, then transported to the laboratory and stored at −80 °C. A 10 g portion of each sample was used for DNA extraction using ISOIL Large Beads ver.2 (NIPPON GENE, Japan). The purity and concentration of the DNA were determined via gel electrophoresis and spectrophotometry. Before pyrosequencing, we amplified the extracted DNA as needed using a GenomiPhi V2 DNA amplification kit (GE Healthcare, USA).

### Metagenome sequencing, de novo assembly and analysis

The extracted genomic DNA was used to create a sequencing library for shotgun pyrosequencing using Roche 454 GS FLX Titanium technology. Raw sequence reads were generated and assembled into contigs using high-quality reads only. We recalled all sequencing reads using the quality recalibration programme Pyrobayes^53^. Obtained sequencing reads were trimmed based on quality, then were de novo assembled using Newbler assembler software (version 2.0). High-quality filtered sequence reads and assembled contigs ≥ 100 bps totalling 1.1 Gbps were used for further analysis.

### Gene prediction and annotation

A local database of all GHs was constructed, which corresponded to selected functional classes: cellulase, EC 3.2.1.4; cellulase 1,4-β-cellobiosidase, EC 3.2.1.91; β-xylosidase, EC 3.2.1.37 and endo-1,4-xylanase, EC 3.2.1.8. The UniProt online database^54^ was used to align the predicted proteome. Assembled contigs were annotated using the gene prediction software Orphelia^55^ with default parameters. Predicted and annotated GH sequences of ORFs were then aligned to the local database using BLASTP (ver. 2.2.18) with a cutoff E value of <10^−20^.

The 454-sequencing approach is prone to producing apparent frame-shift errors via the erroneous insertion (overcall) or deletion (undercall) of extra bases^56^. We compensated in-frame stop codons and frame shifts arising from sequencing errors using the GeneWise programme^57^. The 2  kb-segmented contigs were then aligned to the local database using BLASTX. The complete coding regions of the GHs could then be predicted using GeneWise. The GH family for each region was also identified using Pfam HMMs^58,59^.

### Cloning and protein expression

Nucleotide sequences of the putative CBHs were used for subsequent gene cloning and protein expression in *E. coli* without any codon optimisation. To amplify the catalytic domains from the metagenomic DNA, PCR primers were designed without putative signal peptides, carbohydrate-binding modules and Pro/Thr/Ser-rich linker sequences using the SignalP 3.0 server^60^.

Amplicons were purified using a QIAquick kit (Qiagen), then were cloned into the pET101/D-TOPO vector (Life Technologies) and transformed into competent *E. coli* One Shot TOP10 (Invitrogen) cells. The presence of an insert was verified by colony PCR, and positive clones were grown at 37 °C overnight in liquid LB containing 100 mg/L ampicillin. Plasmids were then isolated using a Wizard plus SV Miniprep DNA Purification System (Promega) and were subjected to Sanger sequencing (Applied Biosystems 3730 DNA Analyzer, Life Technologies).

For protein expression, plasmids containing the amplified genes were transformed into competent *E. coli* BL21 (DE3) Star cells (Life Technologies) or Rosetta-gami B (DE3) pLysS cells (Merck). Transformed cells were grown in an LB medium containing 100 mg/L ampicillin at 37 °C, to an absorbance at 600 nm of ~0.2–0.8 and expression was induced with 0.1 mM isopropyl-β-D(-)-thiogalactopyranoside (IPTG). Growth was then allowed to continue for 5–20 h. Cells were harvested by centrifugation at 1000 × *g* for 10 min. The pellet was resuspended in 50 mM Tris-HCl (pH 8.0) and cell lysis was performed via sonication using an Astrason 3000 instrument (Misonix). Cell debris was removed via centrifugation (16,000 × *g*, 45 min, 4 °C), and the soluble fraction was used as a crude extract for testing the activity of the proteins, with PSA as substrate. The total protein yield for the WT enzyme (RA variant) was 10–20 mg pure protein from 3 L of culture.

### Mutagenesis and expression

Mutagenesis was carried out using the QuikChange Site-Directed Mutagenesis Kit and the QuikChange Lightning Multi Site-Directed Mutagenesis Kit (Agilent Technologies). Primers were designed so that the mutation was close to the middle of the primer, with 10–15 complementary bases on either side of the altered base(s). Mutant plasmids were cloned into *E. coli* XL1-Blue super competent cells or XL10-Gold ultra-competent cells (Stratagene), and DNA was isolated using the Wizard plus SV Miniprep DNA Purification System (Promega), as described above. Site-directed mutant constructs were verified by sequencing both strands using a 3730 Sanger sequencer (Life Technologies).

### PCR cloning of natural variants

Natural variants of HmCel6A were obtained by PCR cloning using forward (5′-CACCATGTTGGACAATCCATTCATCGGAG-3′) and reverse (5′-TTAGGGTTGGATCGGCGGATAG-3′) primers designed based on the sequence of hyper-thermostable cellobiohydrolase HmCel6A (Genbank ID: LC163905), with the four bp sequence CACC added at the 5′-terminus for cloning into pET101/D-TOPO vectors. Template DNA was extracted from metagenome samples collected from a hot spring (33 °C, pH 7.3) in Miyagi, Japan, in 2012. The results are shown in Table S4.

### Purification of enzymes

*E. coli* soluble extracts containing the expressed catalytic domains were used to determine enzyme properties. Culture supernatants were purified via ion-exchange chromatography using HiTrap Q HP columns (GE Healthcare), with proteins loaded in 50 mM Tris-HCl (pH 8.0) and eluted with a gradient of sodium chloride (from 0 to 500 mM).

Eluted fractions exhibiting CBH activity were pooled and concentrated by exchanging 50 mM Tris-HCl (pH 8.0) and 750 mM ammonium sulphate, using a Vivaspin-20 ultrafiltration membrane (Sartorius Stedim Japan, Tokyo, Japan) with a 10 kDa molecular weight cut-off. Proteins were further purified by hydrophobic interaction chromatography using a HiTrap Phenyl HP column (GE Healthcare), with proteins loaded in 50 mM Tris-HCl (pH 8.0) and eluted with ammonium sulphate (gradient from 0 to 750 mM).

Eluted fractions exhibiting CBH activity were pooled and concentrated 10- to 20-fold using the Vivaspin 20 membrane, and then were purified via gel filtration using a HiLoad26/60 Superdex 200 column (GE Healthcare) in 50 mM Tris-HCl (pH 8.0) and 150 mM NaCl. Purified proteins were subjected to a buffer exchange, then concentrated using the Vivaspin-20 membrane to yield a final concentration of 10–20 mg/ml. Proteins were diluted to 1 mg/ml in 50 mM Tris-HCl (pH 8.0) for enzyme activity assays. The purity of all protein preparations was checked and verified by SDS-PAGE and western blot.

### Cellobiohydrolase assays

CBH activity was measured by determining the sugar reduction content using the dinitrosalicylic acid method^61^. D-glucose was used as a standard with pH 5.5 at 30–99 °C using PSA as a substrate. Standard solutions containing 100 μl of 1% PSA, 50 μl of 200 mM sodium acetate (pH 5.5) and 50 μl of crude extract containing the expressed protein were mixed and incubated at 30–99 °C for 20 min. PSA was prepared using a previously described protocol^62^.

To map the optimum temperature and pH, solutions containing 100 μl of 1% PSA, 50 μl of McIlvaine (citrate-phosphate) buffer (pH 3–8), 10 μl of 1 mg/ml purified protein and 40 μl of deionised water were mixed and incubated at 30–99 °C to determine enzyme activity. The results are shown in Fig. S1.

To measure the optimum temperature at optimal pH, solutions containing 100 μl of 1% PSA, 50 μl of sodium acetate (pH 5.5), 10 μl of 1 mg/ml purified protein and 40 μl of deionised water were mixed and incubated at 30–99 °C. Data represent mean values of three independent experiments. The results are shown in Fig. 2a. Furthermore, the effect of metal ions was evaluated by adding the solutions of various metal salts instead of the deionised water (Fig. S3). All the ions were added as chloride salts and the final concentration was adjusted with 1 mM each.

GH activity against polysaccharides was assayed by incubating dilute enzymes at a concentration of 0.05 mg/ml in an assay mixture containing 0.5% (w/v) carboxymethyl cellulose (CMC; Sigma), 0.5% (w/v) Avicel, 0.5% (w/v) PSA, 0.5% (w/v) Lichenan (MP Biomedicals), 0.5% (w/v) Laminarin (Laminaria Digitata, Sigma) or 0.5% (w/v) Xylan (Beechwood, Sigma) in 50 mM sodium acetate (pH 5.5) at 50 °C for 20 min under constant shaking (1400 rpm, Eppendorf Thermomixer). At the end of the 20 min incubation, 200 μl of 3,5-dinitrosalicylic acid (DNS) reagent^61^ was added to the reaction mixture and incubated at 100 °C for 5 min. Absorbance at 540 nm was then measured to determine the amount of reducing sugars were released across the tested temperature and pH ranges. One international unit (U) corresponds to the production of reducing sugars at 1 μmol/min. Specific activities are given as units per milligram of protein (U/mg). The results are shown in Table S3.

### Melting temperature (*T*_m_) analysis using a protein thermal shift assay

A protein thermal shift assay^39,63^ was used to measure the thermostability of the enzymes. Purified proteins were subjected to gradually increasing temperature, and *T*_m_ was measured by mixing 2 μl of 100-fold diluted SYPRO Orange (Life Technologies), 1 μl of 1 mg/ml protein, 5 μl of 200 mM sodium acetate buffer (pH 5.5) and 12 μl of deionised water in the wells of a 96-well, thin-wall PCR plate. The effect of calcium ion was also evaluated by additions of relevant concentrations of CaCl_2_ and EDTA solutions instead of the deionised water. Plates were sealed and heated in a Real Time PCR Detection System (Bio-Rad) from 4 to 100 °C in increments of 0.5 °C. The wavelengths for excitation and emission were 490 and 575 nm, respectively. To calculate *T*_m_ values, the peak of the first derivative was determined using PCR software packages, and the triplicate measurements made for each sample were averaged. The results are shown in Fig. 2b.

### Crystallographic analysis

Crystallisation conditions were screened at 20 °C using the sitting-drop vapour-diffusion method. The drops were comprised of equal amounts (2 μl) of 15–18 mg/ml protein solution and reservoir solution. The crystals for HmCel6A and its complex with cellotriose (Glc3) grew in a reservoir solution containing 20% (w/v) PEG1000, 0.2 M calcium acetate and 0.1 M imidazole buffer (pH 8.0) of Wizard screen I #12 (Emerald BioStructures, Inc.), whereas those for HmCel6A-3SNP grew in 20% (w/v) PEG 1000, 0.2 M lithium sulphate and 0.1 M phosphate-citrate buffer, pH 4.2 (Wizard screen I #39). The solution for cellotriose complex crystals was added to 0.5 mM substrate. For the inactive mutant (D140A) with cellohexaose (Glc6), the crystallisation solution containing 20% PEG 1000, 0.1 M sodium cacodylate (pH 6.5), 0.2 M magnesium chloride (Wizard screen 2 #44) and 0.5 mM substrate was added to the protein solution. Crystals appeared after 3–7 days, and reached a maximum size after 2–3 weeks. Prior to data collection, crystals were mounted using the HAG method^64^, and then flash-cooled at 100 K. Diffraction data were collected at SPring-8 BL38B1. The crystal structures of HmCel6A were determined by molecular replacement with *Humicola insolens* Cel6A (PDB-ID: 1OC5) as the template structure using the MOLREP programme^65^ in the CCP4 package^66^. All the structures were refined as shown in Table S6 using phenix.refine^67^ and COOT^68^, respectively. All structural figures were prepared using PyMol^69^.

### Surface plasmon resonance analysis of substrate binding

The binding affinity of the oligosaccharides to the enzymes for the WT and D140A were analysed using the ProteOn XPR36 System (Bio-Rad Laboratories). Each enzyme was immobilised onto a ProteOn GLH sensor chip with a flow rate of 30 μl/min at 25 °C. All six channels were activated for 5 min using a 150 μl solution of 0.2 M ECD and 0.05 M sulfo-NHS, followed by an immediate injection of 150 μl of 50 μg/ml protein in 10 mM sodium acetate (pH 4.0). Finally, 150 μl of 1 M ethanolamine hydrochloride (pH 8.5) was injected to deactivate any remaining activated carboxyl groups. To evaluate substrate binding, 60 μl of analyte was added in the ProteOn fluidics using a flow rate of 50 μl/min for 60 sec. For channels 1–5, analytes were injected with five different concentrations as follows: Glc2: 1, 0.5, 0.25, 0.125, 0.0625 mM; Glc3: 1, 0.2, 0.04, 0.008, 0.0016 mM; Glc4, Glc5, Glc6: 100, 20, 4, 0.8, 0.16 μM and PSA: 101, 50.5, 25.2, 12.6, 6.3 μM. Running buffer was used with 50 mM acetate (pH 5.5), 100 mM NaCl and 5% (v/v) DMSO. The data were analysed using ProteOn Manager software (Bio-Rad). Binding curves were fitted using the Equilibrium Analysis model of the software.

### Reporting summary

Further information on research design is available in the Nature Research Reporting Summary linked to this article.

## Supplementary information

Supplementary Information

Description of Additional Supplementary Files

Supplementary Data 1

Reporting Summary

## Data Availability

The sequences of the metagenome have been deposited in DDBJ Sequence Read Archive maintained by the DNA Data Bank of Japan under accession numbers of DRA005406. Coordinates and structure factors have been deposited in the Protein Data Bank with accession numbers 6K52, 6K53, 6K54 and 6K55 for HmCel6A, its 3SNP variant, HmCel6A complexed with Glc3 and D140A mutant complexed with Glc6, respectively. All source data for Figs. 2a, 2b, 5a, 5b and Supplementary Figs. 1, 3, 5, 7 are available in Supplementary Data 1.
