# Supplementary material for: Metagenomic mining and structure-function studies of a hyper-thermostable cellobiohydrolase from hot spring sediment
Source: Commun Biol. 2022 Mar 22;5:247. doi: 10.1038/s42003-022-03195-1 (PMC8940973; doi:10.1038/s42003-022-03195-1)
Supplement: Supplementary file 1 — Supplementary Information [file 42003_2022_3195_MOESM1_ESM.pdf]

Supplementary Information

Metagenomic mining and structure-function studies of a hyper-thermostable cellobiohydrolase from hot spring sediment

Authors: Migiwa Takeda,[1] Seiki Baba,[2] Jiro Okuma,[1] Yoshitsugu Hirose,[1*] Asuka Nishimura,[1] Masaki Takata,[2,3] Kohei Oda,[1] Daisuke Shibata,[4*] Takashi Kumasaka,[2*] Yasuhiro Kondo[1]

Affiliations:

[1]Honda Research Institute Japan Co. Ltd., Kazusa Incubation Center, 2-1-5 Kazusa-kamatari, Kisarazu, Chiba 292-0818, Japan.

[2]Japan Synchrotron Radiation Research Institute (JASRI), SPring-8, 1-1-1 Kouto, Sayo, Hyogo 679-5148, Japan.

[3]RIKEN SPring-8 Center, 1-1-1 Kouto, Sayo, Hyogo 679-4198, Japan.

[4]Kazusa DNA Research Institute, 2-6-7 Kazusa-kamatari, Kisarazu, Chiba 292-0812, Japan.

*Correspondence to: Takashi Kumasaka (kumasaka@spring8.or.jp), Yoshitsugu Hirose (yoshitsugu_hirose@jp.honda), Daisuke Shibata (shibata@kazusa.or.jp)

This PDF includes:

Figs. S1−S7
Tables S1−S6
Additional References 70−80

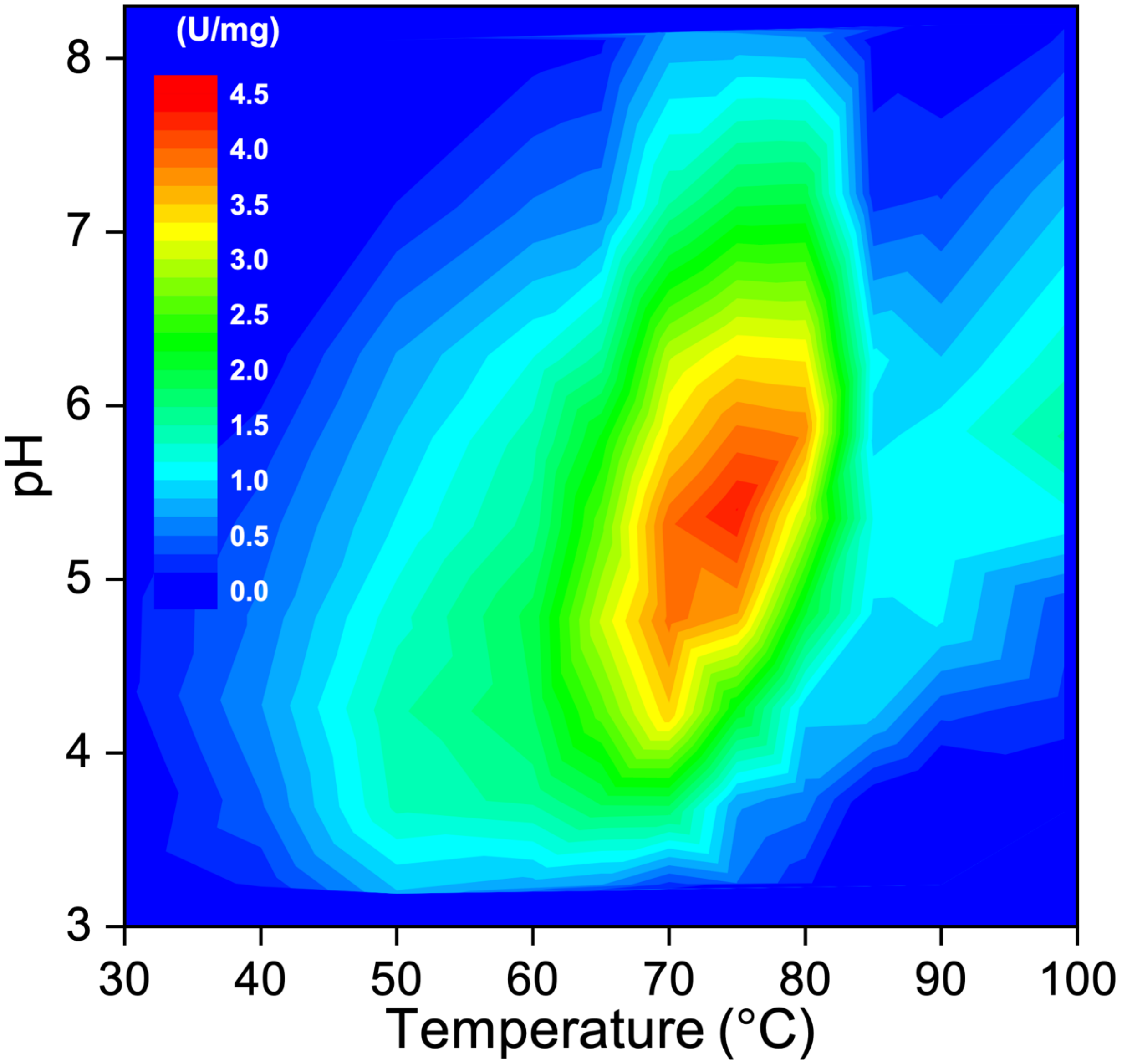

Fig. S1.

Temperature- and pH-dependent specific activity of HmCel6A based on the hydrolysis of phosphoric acid-swollen Avicel (PSA). The activity at each point was measured in triplicate. Samples were at 30–99°C and pH 3–8 using a citrate-phosphate (McIlvaine) buffer. The specific activity (U/mg) is defined as 1 μmol of reduced sugar production/min/mg of purified enzyme.

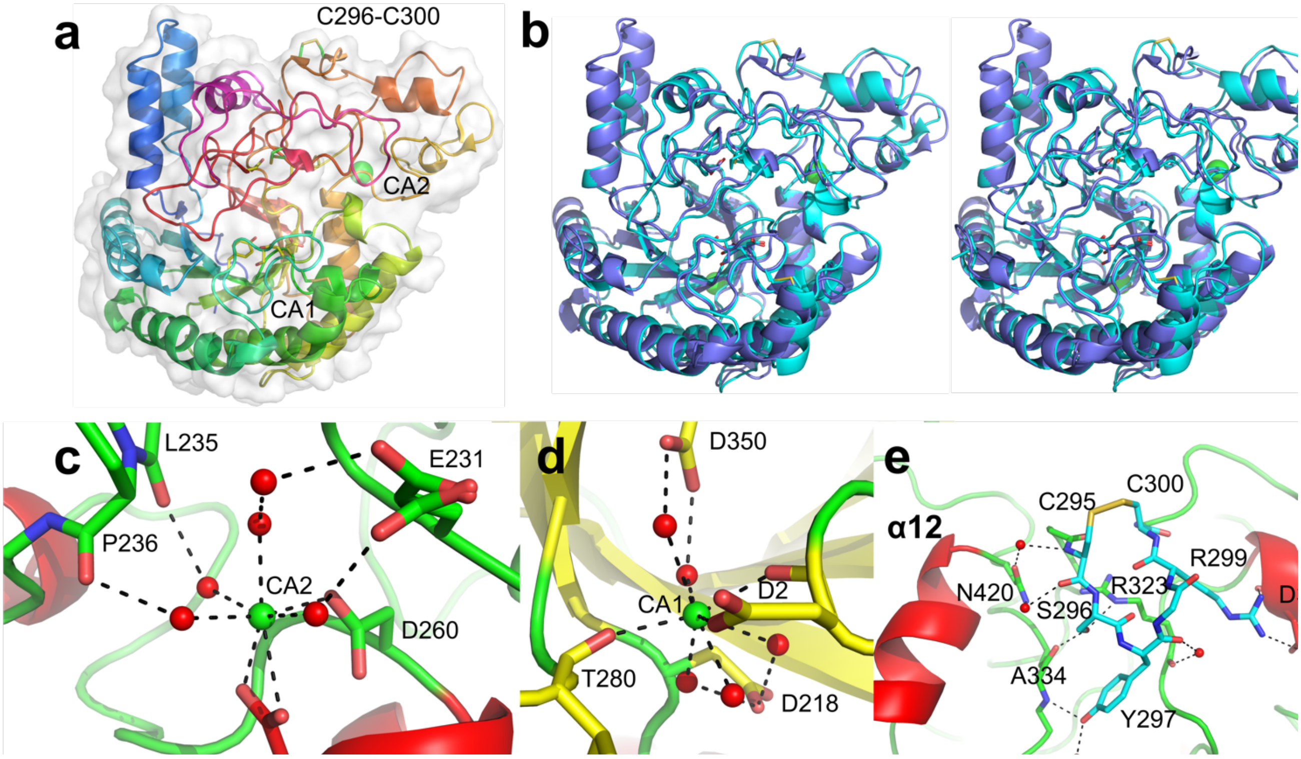

Fig. S2

Crystal structure of HmCel6A. (a) Ribbon representation of HmCel6A, rotated to show the active site loops. (b) Stereo view of HmCel6A (purple) superposed on TfCel6B (cyan). (c) The CA2 calcium site unique to HmCel6A. (d) The CA1 calcium site shared with TfCel6B and other related enzymes. (e) The unique disulphide and six-residue ring structure in HmCel6A.

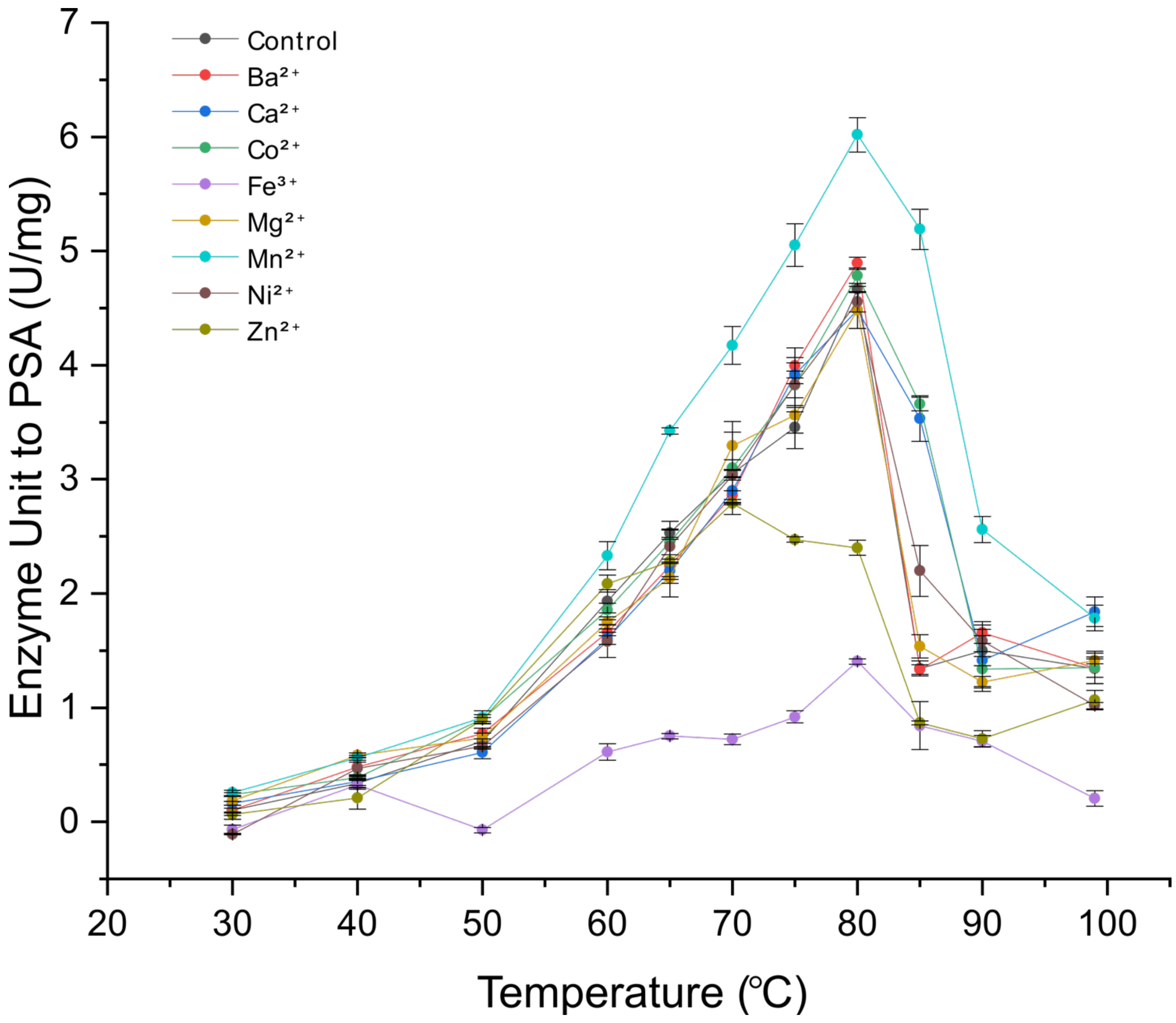

Fig. S3

Effect of metal ions to enzyme activity. Enzyme activities of HmCel6A WT to PSA were measured with the concentration of 1 mM metal ion each. The standard errors are given in bars (n = 3).

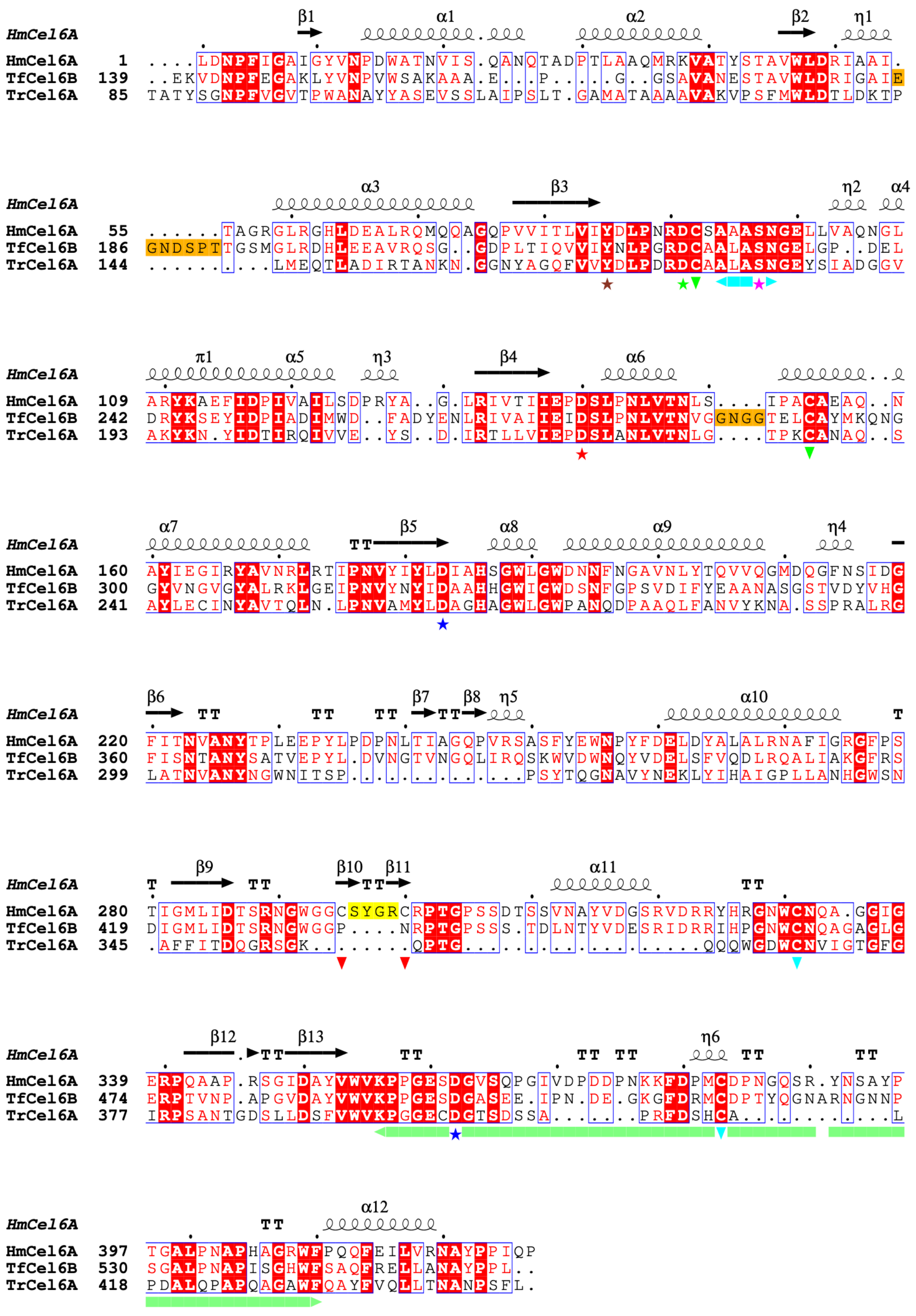

Fig. S4

Structure-based sequence alignment of HmCel6A, TfCel6B and TrCel6A. Catalytically important residues are indicated with coloured stars. Downward triangles indicate pairs of disulphides in HmCel6A. The regions with orange background indicate the gatekeeping loops located at entry or exit of the substrate binding tunnel; the first region shows the exit loop, while the second region shows the entry loop. The six-residue ring formed by the novel disulphide in HmCel6A is indicated by a yellow

background. The active site loop (N-terminal loop, cyan bar), and the bottom loop (C-terminal loop, lime bar) are also shown. The figure was drawn using DSSP[70], MAFFTASH[71], and ESPript 3[72].

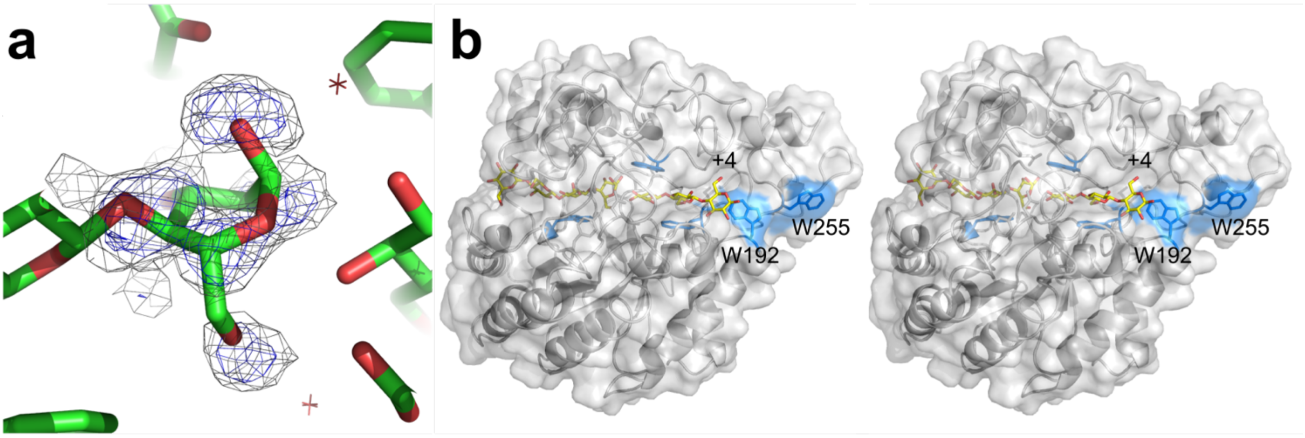

Fig. S5

Substrate binding and recognition. (a) A skewed saccharide structure at the −1 subsite. This structure was observed in chain A of the Glc3-enzyme (wild-type) complex. Omit F_O−F_C map for the corresponding saccharide was contoured with 3σ (grey) and 5σ (blue). (b) Stereo view showing locations of Trp192 and Trp255. Both residues (blue) are located at the entrance side of the active site tunnel. Trp192 forms the subsite +4.

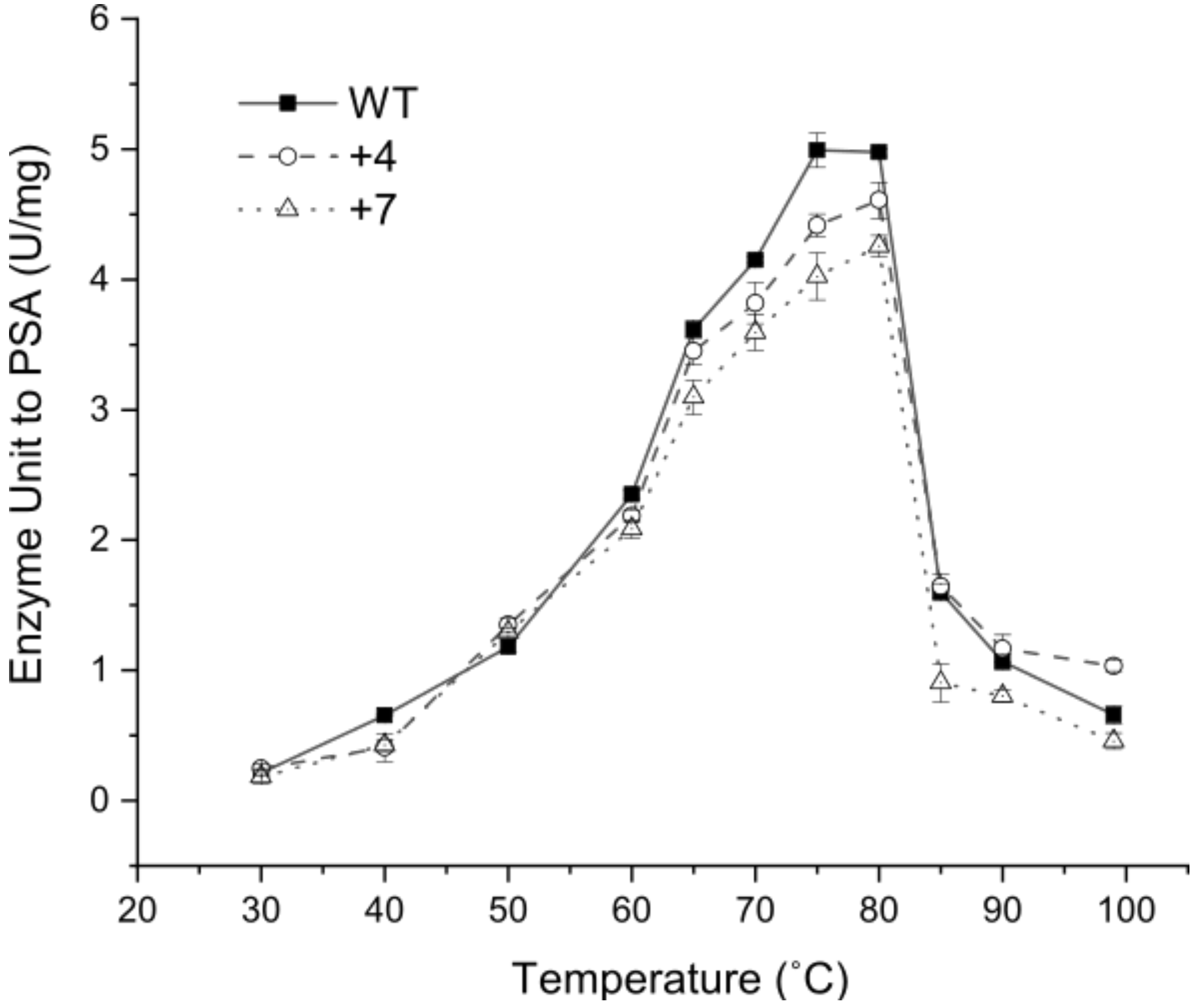

Fig. S6

Effect of exit loop extensions on temperature-dependent specific activity on PSA substrate; WT: HmCel6A Wild Type (RA); +4: insertion of four amino acids (NDSP); and +7: insertion of seven amino acids (NDSPTTG). The data are the mean values of each independent experiment. The standard errors are given in bars (n = 3).

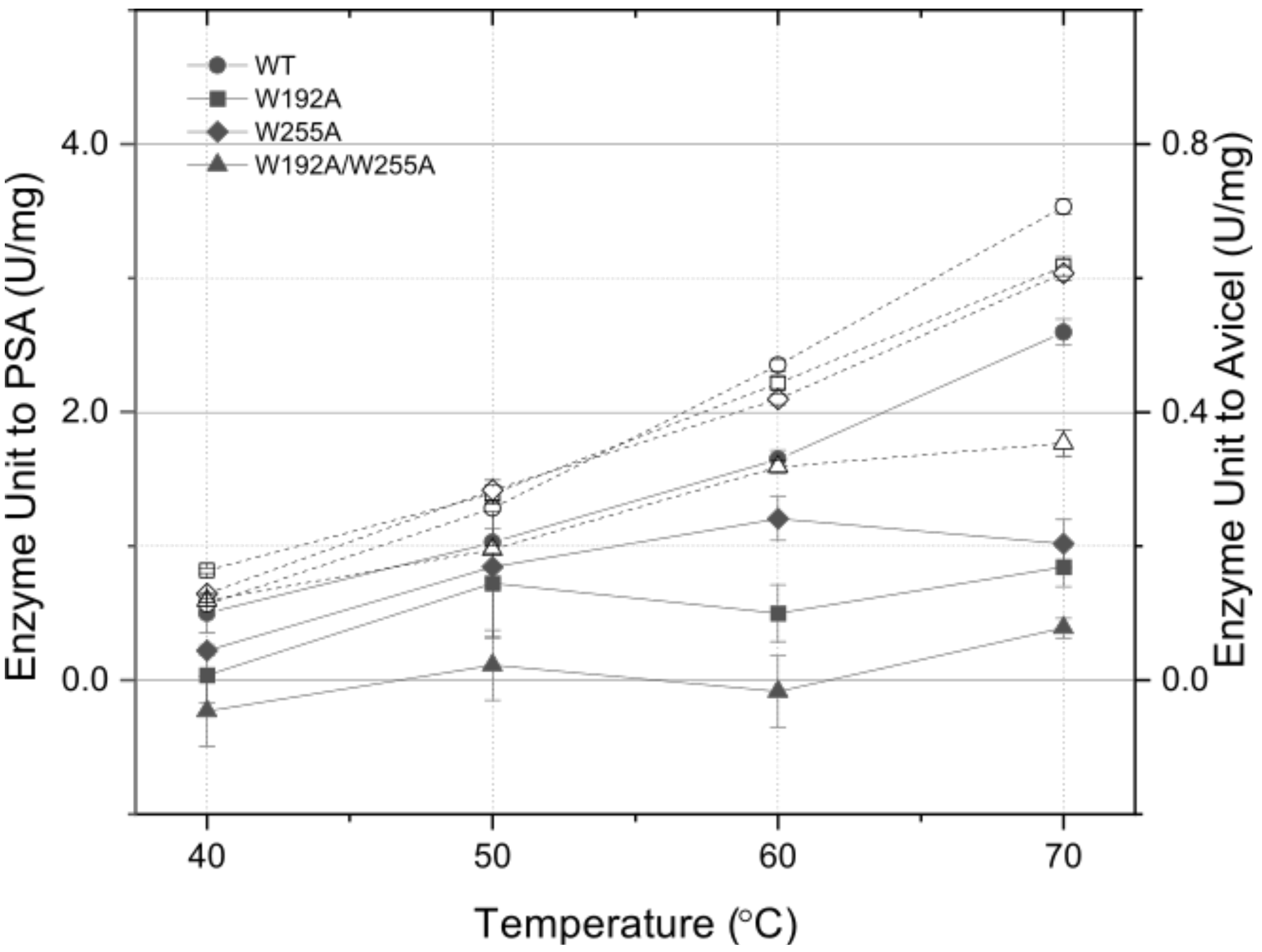

Fig. S7

Effect of tryptophans, W192 and W255. Enzyme activities of HmCel6A WT for Avicel (solid line) and PSA (broken line) are shown.

Table S1.

Summary of statistics for the hot spring microbial community metagenome AR19

Characteristic	Amount
Number of 454 pyrosequencing runs	3
Number of raw reads generated	2,766,332
Raw bases generated (bp)	1,106,217,617
Average read length (bp)	399.9
Assembly statistics	
Number of assembled contigs	101,372
Largest contig size (bp)	187,970
Average contig size (bp)	1,027
Total assembled contig length (bp)	104,096,316
Number of reads completely or partially assembled	2,040,651
Number of singletons*1	596,706
Total singleton length (bp)	225,926,617
Number of contigs and singletons	698,078
Total contig and singleton length (bp)	330,022,933
Phylogenetic assignment against the KEGG database*2	
Bacterial content (bp)	59,887,880
Archaeal content (bp)	3,057,656
Eukaryotic content (bp)	25,499
Viral content (bp)	384,255
Unclassified content (bp)	266,667,643

*1 Reads that could not be assembled were assigned as singletons.

*2 Phylogenetic assignment of all contigs and singletons was performed using BLAST and by comparison against the KEGG database to classify them into bacterial, archaeal, eukaryotic, viral, or unclassified sets.

Table S2.

Carbohydrate-active enzyme (CAZy) families of Pfam-annotated endoglucanases (EGs) and cellobiohydrolases (CBHs) in the hot spring metagenome AR19

CAZy family	Pfam domain	Number of ORFs	Ratio*
GH5	PF00150	46	3.7%
GH6	PF01341	2	0.2%
GH7	PF00840	0	0.0%
GH9	PF00759	15	1.2%
GH44	PF12891	5	0.4%
GH45	PF02015	0	0.0%
GH48	PF02011	7	0.6%
Total		75	6.1%

*The ratio of the number of cellulases in each CAZy family to the total number of GHs annotated is indicated as a percentage.

Table S3.

Carbohydrolytic activity of HmCel6A

Substrates	Specific activity[a] (U/mg)	Relative activity[b]
PSA	1.81 ± 0.10	-
Avicel (2 hr)	0.08 ± 0.05	4.4%
Lichenan	0.27 ± 0.02	14.9%
Laminarin	0.02 ± 0.01	1.1%
CMC	N.D.	0.0%
Xylan	0.01 ± 0.02	0.6%

[a] The incubation time for activity measurement was 20 min (with all substrates except Avicel). Measurements were performed in triplicate and standard errors calculated. PSA, phosphate-swollen Avicel; CMC, carboxymethyl cellulose.
[b] Activities shown are relative to that measured using PSA as the substrate.

Table S4.

List of mutants obtained by colony PCR. The sediment samples were collected from Onikobe-Jigokudani geothermal area (38˚48'N, 140˚40'E), Miyagi prefecture, Japan.

	Metagenome DNA samples					
	AR19	OJS2	OJS4	OJS7	OJS9	Total
No. of clones amplified by colony PCR	240	48	48	48	48	432
No. of clone hits						
RA (R299/A351)*	4	0	0	0	0	4
QA (Q299/A351)	18	3	4	3	1	29
QV (Q299/V351)	1	0	0	0	0	1
RA/E254D	1	0	0	0	0	1
RA/P88S/L230F/F414S†	0	2	0	0	0	2
QA/A33V	2	0	0	0	0	2
QA/T228I	0	0	1	0	0	1
QA/E254G	0	0	0	1	0	1
QA/R269C	1	0	0	0	0	1
QA/S306G	2	0	0	0	0	2
QA/I84T/A406T	0	0	0	1	0	1
QA/R166H/E360K	0	0	1	0	0	1
Total no. of clone hits	29	5	6	5	1	46
Temperature of sample	70.3	33.2	40.5	77.2	63.5	
pH of sample	7.2	7.3	7.6	8.0	7.1	
Sampling year	2009	2012	2012	2012	2012	

*: This variant is defined as wild type.

†: This variant is defined as 3SNP.

Table S5.

Comparison of enzymatic and structural properties of GH6 cellobiohydrolases

	T_m (°C)	T_{opt} (°C)	PDB-ID	# total residues, atoms	# hydrogen bonds: between all[*4], side-chains (#networks)[*5]	# Salt bridges, atoms, residues[*6]	# disulphides, # metals	TKSA[*7] ΔG_{elec} (kJ/mol)	Hydrophobic cluster[*5]: Area (Å^2), # clusters, # contacts in the largest cluster
HmCel6A	85.5	80	6K52	428, 3,435	319, 49 (28)	28, 14	3, 2	-358.7	8888.5, 12, 139
HmCel6A-3SNP	96	95	6K53	428, 3,360	318, 47 (28)	28, 14	3, 0	-345.5	8885.3, 9, 145
TfCel6B	65[49]	60[49]	4B4H	420, 3,215	302, 45 (27)	17, 12	2, 1[*1]	-275.6	6943.7, 8, 98
CfCel6B	n/a	n/a	7CBD	443, 3,348	332, 45 (24)	24, 16	2, 0	-300.9	6710. 5, 11, 97
XooCbsA	n/a	n/a	5XYH	424, 3,217	318, 38 (23)	19, 13	2, 0	-284.7	7398.7, 10, 114
HjCel6A	60.2 ± 0.4[27]	55[27]	1CB2	363, 2,746	273, 45 (15)	16, 9	2, 0	-161.5	4907.1, 9, 91
CtCel6A	62.6 ± 0.4[27]	60[27]	4A05	360, 2,793	275, 35 (16)	22, 14	2, 0[*2]	-230.1	4373.9, 9, 61
CcCel6A	n/a	n/a	3VOG	362, 2,800	279, 38 (20)	21, 13	2, 0	-279.7	6783.2, 7, 88
CcCel6C	n/a	n/a	3A64	371, 2,894	286, 43 (28)	21, 14	2, 0[*3]	-281.0	7152.7, 8, 113
PcCel6A	55[75]	n/a	5XCY	358, 2,709	280, 34 (21)	9, 5	2, 0	-119.4	7302.5, 7, 107
HiCel6A	65.2 ± 0.1[27]	65[27]	1BVW	360, 2,829	275, 44 (23)	23, 14	2, 0[*3]	-270.5	5853.7, 10, 91
HJPlus	71.9 ± 0.6[27]	70[27]	4I5R	364, 2,777	268, 29 (18)	16, 10	2, 0	-206.4	4865.2, 8, 85
3C6P	80.1±0.4[27]	75[27]	4I5U	357, 2,833	271, 27 (15)	20, 12	2, 0	-205.5	5696.6, 9, 92

Abbreviations: TfCel6B, *Thermobifida fusca* Cel6B[23]; CfCel6B, *Cellulomonas fimi* Cel6B (formerly CbhA)[42]; XooCbsA, *Xanthomonas oryzae* pv. *oryzae* CbsA, HjCel6A, *Hypocrea jecorina* (*Trichoderma reesei*) Cel6A[40]; CtCel6A, *Chaetomium thermophilum* Cel6A[73]; CcCel6A, *Coprinopsis cinerea* Cel6A[74]; CcCel6C, *Coprinopsis cinerea* Cel6C[51]; PcCel6A, *Phanerochaete chrysosporium* Cel6A[75,76], HiCel6A, *Humicola insolens* Cel6A[41]; HJPlus and 3C6P, Chimeric Cel6 constructed from cellulases of *Humicola insolens*, *Hypocrea jecorina* and *Chaetomium thermophilum*[27]; HmCel6A and HmCel6A-3SNP (this study).

*1: Although 4B4H (apo-enzyme) does not contain any calcium, 4B4F (substrate complex) has two Ca ions, one of which is coordinated similarly to CA1 in HmCel6A.

*2: One Li ion interacts with two sugar molecules at the active centre.

*3: One Mg ion is present at the surface, and bound via water-mediated interactions.

*4: Calculated using Volume, Area, Dihedral Angle Reporter (VADAR) software[77]. The number of residues with hydrogen bonds and its ratio against the total number of residues were shown.

*5: Calculated using Hydrophobic clusters in ProteinTools server[80].

*6: Calculated as atom based using the ESBRI (Evaluating the Salt BRIdges in proteins) server[78]. Only the unique pairs are shown.

*7: Calculated using the TKSA-MC (Tanford-Kirkwood Surface Accessibility model with the Monte Carlo method) server[79].

n/a, data not available.

The A chain of each PDB data was applied to all the calculations.

Table S6.

Crystallographic data collection and refinement statistics for HmCel6A

	Wild type (Ca^{2+}-bound form)	3SNP (P88S/L230F/F414S)	Wild type (Glc_3 complex)	D140A (Glc_6 complex)
PDB entry	6K52	6K53	6K54	6K55
Data collection				
Space group	*H*32 (*R*32)	*H*32 (*R*32)	*C*2	*C2*
Cell parameters (Å, °)	*a* = 141.2,	*a* = 141.9,	*a* = 170.6, *b* = 138.7,	*a* = 173.9, *b* = 140.0,
	c = 223.2	*c* = 224.1	*c* = 110.6, β = 102.0	*c* = 110.6, β = 101.1
Resolution range (Å)[a]	50.00–1.68	50.00–1.89	50.00 - 1.905	50-2.883
(Outmost shell)	(1.74–1.68)	(1.96–1.89)	(1.973 - 1.905)	(2.986-2.883)
No. of observed reflections	1,587,376	732,046	1,288,532	243,905
No. of unique reflections	97,032	69,369	194,529	56,407
Redundancy[a]	16.3 (15.7)	10.6 (10.2)	6.6 (6.0)	4.3 (2.8)
Completeness (%)[a]	100.0 (100.0)	100.00 (100.00)	99.2 (94.4)	96.0 (80.2)
R_{merge} (%)[ab]	7.2 (60.2)	7.7 (57.9)	12.7 (100.)	19.9 (82.6)
Mean $I/\sigma(I)$[a]	36.7 (6.6)	28.1 (5.3)	17.8 (2.4)	5.7 (1.2)
Wilson *B* factor (Å^2)	17.4	20.4	21.6	67.0
Refinement				
R_{work} (%)/ R_{free} (%)	17.4/19.6	16.0/18.9	17.6/20.1	17.2/21.9
No. of atoms				
Protein/Waters/Ligands	3434/601/11	3346/687/15	9997/1552/249	9941/92/240
Average *B* factor (Å^2)				
Protein/Waters/Ligands	19.3/33.1/22.4	20.3/34.6/44.0	22.9/35.0/29.5	56.1/49.7/57.0
Bond lengths (Å)	0.007	0.007	0.007	0.009
Bond angles (°)	1.13	1.06	0.85	1.03
Clashscore	1.8	1.1	1.8	5.9
Ramachandran favoured/outliers (%)	97/0	97/0	97/0	93.9/0.2

[a] Values in parentheses are those for the highest resolution shell.
[b] $R_{merge} = \Sigma_{hkl}\Sigma_i|I_i-<I>|/\Sigma_{hkl}\Sigma_i |I|$.

Additional References

70. Touw, W. G. *et al.* A series of PDB related databases for everyday needs. *Nucleic Acids Res.* **43**, D364-D368 (2015). https://dx.doi.org/10.1093%2Fnar%2Fgku1028

71. Kinjo, A. R. *et al.* Protein Data Bank Japan (PDBj): maintaining a structural data archive and resource description framework format. *Nucleic Acids Res.* **40**, D453-D460 (2012). https://doi.org/10.1093/nar/gkr811

72. Robert, X. & Gouet, P. Deciphering key features in protein structures with the new ENDscript server. *Nucleic Acids Res.* **42**, W320-W324 (2014). https://doi.org/10.1093/nar/gku316

73. Thompson, A. J. *et al.* Structure of the catalytic core module of the *Chaetomium Thermophilum* family GH6 Cellobiohydrolase Cel6A. *Acta Crystallogr. D* **68**, 875-882 (2012). https://doi.org/10.1107/s0907444912016496

74. Tamura, M., Miyazaki, T., Tanaka, Y., Yoshida, M., Nishikawa, A. & Tonozuka, T. Comparison of the structural changes in two cellobiohydrolases, CcCel6A and CcCel6C, from *Coprinopsis cinerea* - a tweezer-like motion in the structure of CcCel6C. *FEBS J.* **279**, 1871-1882 (2012). https://doi.org/10.1111/j.1742-4658.2012.08568.x

75. Tachioka, M. et al. Crystal structure of a family 6 cellobiohydrolase from the basidiomycete *Phanerochaete chrysosporium*. *Acta Crystallogr. F* **73,** 398-403 (2017). https://dx.doi.org/10.1107%2FS2053230X17008093

76. Heinzelman, P. *et al.* SCHEMA recombination of a fungal cellulase uncovers a single mutation that contributes markedly to stability. *J. Biol. Chem.* 284, 26229-26233 (2009). https://dx.doi.org/10.1074%2Fjbc.C109.034058

77. Willard, L. *et al.* VADAR: a web server for quantitative evaluation of protein structure quality. *Nucleic Acids Res.* **31**, 3316-3319 (2003). https://doi.org/10.1093/nar/gkg565

78. Costantini, S., Colonna, G. & Facchiano, A. M. ESBRI: a web server for evaluating salt bridges in proteins. *Bioinformation* **3**, 137-138 (2008). https://doi.org/10.6026/97320630003137

79. Contessoto, V.G., de Oliveira, V.M., Fernandes, B.R., G Slade, G.G., Leite, V.B.P. TKSA-MC: A web server for rational mutation through the optimization of protein charge interactions. *Proteins* **86,** 1184-1188 (2018). https://doi.org/10.1002/prot.25599

80. Ferruz, N., Schmidt, S., Höcker, B. ProteinTools: a toolkit to analyze protein structures. *Nucl. Acids Res.* **49,** W559-W566 (2021). https://doi.org/10.1093/nar/gkab375
