# Supplementary material for: Metagenomic mining and structure-function studies of a hyper-thermostable cellobiohydrolase from hot spring sediment
Source: Commun Biol. 2022 Mar 22;5:247. doi: 10.1038/s42003-022-03195-1 (PMC8940973; doi:10.1038/s42003-022-03195-1)
Supplement: Supplementary file 4 — Reporting Summary [file 42003_2022_3195_MOESM4_ESM.pdf]

nature research

Corresponding author(s): Takashi Kumasaka, Daisuke Shibata, Yoshitsugu Hirose

Last updated by author(s): Jan 15, 2021

Reporting Summary

Nature Research wishes to improve the reproducibility of the work that we publish. This form provides structure for consistency and transparency in reporting. For further information on Nature Research policies, see our Editorial Policies and the Editorial Policy Checklist.

Statistics

For all statistical analyses, confirm that the following items are present in the figure legend, table legend, main text, or Methods section.

n/a	Confirmed	
☐	☒	The exact sample size (*n*) for each experimental group/condition, given as a discrete number and unit of measurement
☐	☒	A statement on whether measurements were taken from distinct samples or whether the same sample was measured repeatedly
☐	☒	The statistical test(s) used AND whether they are one- or two-sided<br>*Only common tests should be described solely by name; describe more complex techniques in the Methods section.*
☐	☒	A description of all covariates tested
☐	☒	A description of any assumptions or corrections, such as tests of normality and adjustment for multiple comparisons
☐	☒	A full description of the statistical parameters including central tendency (e.g. means) or other basic estimates (e.g. regression coefficient) AND variation (e.g. standard deviation) or associated estimates of uncertainty (e.g. confidence intervals)
☐	☒	For null hypothesis testing, the test statistic (e.g. F, t, r) with confidence intervals, effect sizes, degrees of freedom and P value noted<br>*Give P values as exact values whenever suitable.*
☐	☒	For Bayesian analysis, information on the choice of priors and Markov chain Monte Carlo settings
☐	☒	For hierarchical and complex designs, identification of the appropriate level for tests and full reporting of outcomes
☐	☒	Estimates of effect sizes (e.g. Cohen's *d*, Pearson's *r*), indicating how they were calculated

Our web collection on statistics for biologists contains articles on many of the points above.

Software and code

Policy information about availability of computer code

Data collection	Crystallographic data collection at synchrotron: SPring-8 BSS ver 2012.
Data analysis	Metagenome analysis and gene annotation: Newbler assembler ver. 2.3 or 2.5.3, blastall ver. 2.2.18, GeneWise ver.2, Pfam ver. 23.0, HMMER ver. 2.3, and SignalP ver. 3.0.<br>Crystallographic analysis: PHENIX 1.12_2829/1.15.2_3472, HKL-2000 v716.1, HKLSuite 0.95 & HKL data processing system Version 2.3.12, MOLREP CCP4-7.0

For manuscripts utilizing custom algorithms or software that are central to the research but not yet described in published literature, software must be made available to editors and reviewers. We strongly encourage code deposition in a community repository (e.g. GitHub). See the Nature Research guidelines for submitting code & software for further information.

Data

Policy information about availability of data

All manuscripts must include a data availability statement. This statement should provide the following information, where applicable:

- Accession codes, unique identifiers, or web links for publicly available datasets
- A list of figures that have associated raw data
- A description of any restrictions on data availability

The sequences of the metagenome have been deposited in DDBJ Sequence Read Archive maintained by the DNA Data Bank of Japan under accession numbers of DRA005406. Coordinates and structure factors have been deposited in the Protein Data Bank with accession numbers 6K52, 6K53, 6K54 and 6K55 for HmCel6A, its 3SNP variant, HmCel6A complexed with Glc3 and D140A mutant complexed with Glc6, respectively.

Field-specific reporting

Please select the one below that is the best fit for your research. If you are not sure, read the appropriate sections before making your selection.

☒ Life sciences ☐ Behavioural & social sciences ☐ Ecological, evolutionary & environmental sciences

For a reference copy of the document with all sections, see nature.com/documents/nr-reporting-summary-flat.pdf

Life sciences study design

All studies must disclose on these points even when the disclosure is negative.

Sample size	Sequences of the targeted genes were confirmed by duplicated experiments. No statistical estimation of sample size was performedfor each measurement.
Data exclusions	Data were not excluded from analysis.
Replication	For experiments involving quantification of enzyme activity and substrate binding affinity, n=3 or more was chosen as the minimal replicate number, and sample amount was determined based on each conventional protocol.
Randomization	There was no requirement of randomization in the metagenomic gene finding and purotein characterization, because the aim of this study was to find new enzymes in an geothermal area, but not to characterize the area in terms of metagenomic gene distribution.
Blinding	Blinding was not needless, because our research is not related to a randomized controllled trial but its aim is a discovery of target enzymes having desired properties. Quantifications were performed using analytical and computational pipeline applied equally to all conditions.

Reporting for specific materials, systems and methods

We require information from authors about some types of materials, experimental systems and methods used in many studies. Here, indicate whether each material, system or method listed is relevant to your study. If you are not sure if a list item applies to your research, read the appropriate section before selecting a response.

Materials & experimental systems

n/a	Involved in the study	
☒	☐	Antibodies
☒	☐	Eukaryotic cell lines
☒	☐	Palaeontology and archaeology
☒	☐	Animals and other organisms
☒	☐	Human research participants
☒	☐	Clinical data
☒	☐	Dual use research of concern

Methods

n/a	Involved in the study	
☒	☐	ChIP-seq
☒	☐	Flow cytometry
☒	☐	MRI-based neuroimaging
